# Normal fertilisation rates and serum 25-OHD levels among couples undergoing in-vitro fertilisation: a prospective cohort study

**DOI:** 10.1186/s12884-020-02959-z

**Published:** 2020-06-05

**Authors:** Liu Jiang, Juan Yang, Jianyuan Song, Yajun Hu, Kun Qian

**Affiliations:** 1 Reproductive Medicine Centre, Tongji Hospital, Tongji Medical College, Huazhong University of Science and Technology, Wuhan, China; 2 Reproductive Medicine Centre, First Hospital of Wuhan, Tongji Medical College, Huazhong University of Science and Technology, Wuhan, China; 3 The Fourth Affiliated Hospital, Zhejiang University School of Medicine, Hangzhou, China

**Keywords:** 25-OHD, IVF-ET, Normal fertilisation rate, Couple

## Abstract

**Background:**

The aim of this study was to explore the correlation between serum vitamin D levels in couples undergoing in-vitro fertilisation (IVF) and normal fertilisation process.

**Methods:**

Between March 2016 and March 2017, we performed a prospective cohort study at an academic reproductive medicine centre to measure serum 25-hydroxyvitaminD (25-OHD) levels of 1232 couples before controlled ovarian stimulation. Generalized linear regression and binary multivariate logistic regression were employed to assess whether 25-OHD levels in men and women correlated with normal fertilisation rates and low fertilisation rate (LFR).

**Results:**

Serum 25-OHD levels in women were classified into three groups: Group A, less than 10%; Group B, between 10 and 90%; and Group C, greater than 90%. Using generalized linear regression, we observed that female 25-OHD levels were related to normal fertilisation rates. Adjusted normal fertilisation rates from Group A to Group C in women were 59.50, 62.72, and 66.13%, respectively (*P* = 0.007). After binary logistic regression analysis, for women, compared with Group C, the ORs for LFR were 4.814 in Group A (95% CI, 1.266–18.309, *P* = 0.021) and were 3.204 in Group B (95% CI, 0.949–10.812, *P* = 0.061). Male 25-OHD levels were not related to the probability of low fertilisation rate (*P* > 0.05).

**Conclusions:**

Circulating 25-OHD concentrations in women appear to be associated with normal fertilisation rates and low fertilisation rates in IVF cycles, but not in men. A further randomized controlled trial with vitamin D supplementation is needed to demonstrate whether female vitamin D levels exert an effect on the normal fertilisation process.

**Trial registration:**

https://clinicaltrials.gov/;NCT03305510; Registered 08 October 2017 - Retrospectively registered.

## Background

Vitamin D deficiency is highly prevalent and increasing [1–4] due to reduced outdoor-residence time and increased use of sunlight-avoidance measures [5]. Interestingly non-skeletal actions of vitamin D have recently generated much attention, including in the area of reproductive processes.

Vitamin D was found to be involved in steroidogenesis, follicular development and spermatogenesis. Vitamin D receptor (VDR) and vitamin D synthetase have been identified widely in both female and male reproductive tissues, including Sertoli cells, Leydig cells, and spermatozoa [6–9]. In human ovarian cells, vitamin D elevated estradiol production by 9% and stimulated its production by 60% in synergism with insulin-which may be interpreted as a promotion of aromatase activity [10–13]. In addition, vitamin D exerted direct actions on spermatozoa and activated molecular pathways involved in sperm motility, capacitation, and the acrosome action [9, 14]. Male VDR-knockout male rats exhibited oligoasthenospermia and hypergonadotrophic hypogonadism; and female rats showed irregular oestrous cyclesand decreased fertility [15].

Fertilisation is the rate-determining and key step in in-vitro fertilisation (IVF), with an average rate of 60% [16], and is an intricate, complex, and orderly process. Calcium influx in capacitation, acrosome reaction, and activation of the mature oocyte is essential for fertilisation and the surge in ooplasmic calcium induced by fertilisation is related to activation of the mature oocyte, which is of paramount importance in embryonic embryo development [17, 18]. Also, vitamin D is one of the primary regulators of calcium balance in cells [19]. Several investigators have explored the potential effect of vitamin D on the intracellular calcium influx and the acrosome reaction in vitro [17, 20–22]. Therefore, we hypothesized that vitamin D plays a functional role in the fertilisation process.

The pathogenic contribution to infertility appears to be equivalent between sexes when excluding confounding factors [23], and we have uncovered no reports exploring the association between the serum vitamin D status of couples and fertilisation rates. Therefore, we devised a large prospective cohort study with a 1-year recruitment period to explore this association.

## Methods

### Patient eligibility criteria

Between March 2016 and March 2017, we performed a large prospective, non-interventional, single-center, cohort study and measured plasma concentrations of 25-hydroxyvitaminD (25-OHD) prior to controlled ovarian stimulation in couples who underwent conventional IVF. The inclusion criteria were: females [1] 20–42 of age without decreased ovarian reserve according to Bologna criteria [2], with a baseline follicle-stimulating hormone (FSH) below 10 mIU/ml [3], with a body mass index (BMI) below 30 kg/m^2^, and [4] undergoing their first IVF cycle. Cycles entailing sperm donation (*n* = 160) or frozen oocytes (*n* = 20) were excluded from our study. This study was approved by the Institutional Review Board at Huazhong University of Science and Technology, Wuhan, China. All of the participants provided informed written consent.

### Study design

For the short gonadotropin releasing hormone agonist (GnRH-a) long protocol, 0.1 mg of GnRH-a (Diphereline; Beaufour Ipsen, France) per day was injected from the mid-luteal phase onward. For the GnRH-a prolonged protocol (prolonged protocol), 3.75 mg of GnRH-a (Diphereline; Beaufour Ipsen, France) was injected on day 2 of the menstrual cycle. Down-regulation was confirmed 14 days after pituitary down-regulation for the short GnRH-a protocol and 28 days for the prolonged protocol (resulting in a follicle diameter < 5 mm, hormonal concentrations < 50 pg/ml for estradiol [E2] and < 5 IU/L for luteinizing hormone [LH], and endometrial thickness < 5 mm). Then 75–300 IU of rhFSH (Gonal-F; Serono, Switzerland) per day was injected for approximately 10 days. The dose was based on patient age, ovarian reserve testing, and body weight. For the gonadotropin releasing hormone antagonist (GnRH-ant) protocol, rhFSH (Gonal-F) was injected on day 2 of the menstrual cycle and on day 6, GnRH antagonists (Ganirelix; Merck & Co Inc.) were injected until the human chorionic gonadotropin (HCG) trigger day. When at least three follicles reached 17 mm in diameter, 10,000 IU HCG(Livzon Pharmaceutical Group Inc., China) was used to trigger ovulation, and transvaginal oocyte retrieval was performed 34–36 h later. Fertilisation was achieved using standard IVF. Spermatozoa were treated by density-gradient centrifugation, and the sperm density was adjusted to 10,000 sperm per oocyte. All of the cycles at our center used short-term fertilisation; that is, oocytes were removed after co-incubation with sperm for 3–4 h. Pronuclei were observed and recorded after 16–18 h of fertilisation, And embryo transfer was performed on day 3 after fertilisation. The luteal phase was supported.

To avoid seasonal differences, we excluded couples if the time between serum 25-OHD measurement and ovum pick-up was over a season in length. Mean serum 25-OHD concentrations in each month were calculated, and we concluded that the 25-OHD status remained stable during June to October, November, December to April, and May (data regarding 25-OHD levels in each month are provided in Figs. S1 and S2). Thus, we obtained a full data set that included serum 25-OHD levels and clinical characteristics of 1232 couples.

### Assay of serum the 25-OHD status in couples and grouping

Prior to conducting controlled ovarian hyperstimulation (COH), all of the couples had routine preoperative examinations. We collected 2 ml of blood using lithium heparin, and then performed a 25-OHD assay by liquid chromatography-tandem mass spectrometry. The examination was performed according to standardized protocols and appropriate quality control procedures, including multiple measurements of the same sample (Roche, Mannheim, Germany). The intraassay and interassay coefficients of variation were both below 10%, with a detection range between 3.0 and 70.0 ng/ml. The physicians and biologists engaged in the cycle management were blinded as to the results of the 25-OHD assessment. Manson JA, et al. reported that sufficient 25-OHD level is defined as higher than 16 ng/ml, so as to avoid adverse skeletal damage [24]; however, this definition may not fit as a serum 25-OHD threshold for non-skeletal actions, particularly with respect to fertility in men and women. Therefore, we classified the levels of 25-OHD into three groups: Group A, less than 10%; Group B, between 10 and 90%; and Group C, greater than 90%).

### Definition of normal fertilisation rate

Fertilisation condition was defined as the presence of pronuclei in the retrieved oocytes, 16–18 h after conventional insemination. The normal fertilisation rate was defined as the percentage of 2PNs per the total number of retrieved oocytes. Low fertilisation rate (LFR) was defined as a fertilisation rate less than 20% of normal.

### Statistical analysis

We conducted analyses with SPSS (version 22.0, Chicago, IL, USA), and figures were generated by Graph Pad Prism7 (San Diego, CA, USA). First, we evaluated differences in baseline characteristics and normal fertilisation rates according to different 25-OHD groups. We analysed continuous variables using one-way ANOVA or Kruskall-Wallis H test, as appropriate. Categorical variables with Pearson’s Chi-square test. In addition, we employed generalized linear regression to assess whether 25-OHD levels in men and women correlated with normal fertilisation rates. The distribution of normal fertilisation rate was followed a gamma distribution, and the link function was logarithmic. Normal fertilisation rates were set as the dependent variable, and independent variables included female 25-OHD level, male 25-OHD level, female age, male age, sperm progressive motility rate, BMI, baseline FSH, duration of infertility, having primary infertility or not, stimulation protocol, daily dose of gonadotrophins, and number of mature oocytes retrieved. Male 25-OHD levels, female 25-OHD levels, COH protocol, and having primary infertility or not were categorical variables and converted to dummy variables. Through the regression model, the marginal mean of the dependent variable were obtained, and the differences based on the marginal mean were determined using the Wald Chi-square test. Next, Spearman’s correlation coefficient test was used to test positive or negative linear correlations between female 25-OHD groups and normal fertilisation rate. Finally, binary multivariate logistic regression was employed to assess whether 25-OHD groups correlated with low fertilisation rate. A normal fertilisation rate < 0.2 (with LFR) was set as the reference variable. The variables were then processed in the same way as the generalized linear model described above. All of the tests were two sided, and the significance level was set at 0.05.

## Results

### Baseline characteristics

We recruited 1232 couples who satisfied our inclusion criteria. The mean 25-OHD levels in each month among men and women are illustrated in Figs. S1 and S2, in which we classified 12 months into four seasons: June to October, November, December to April, and May. There were no statistical differences among mean 25-OHD levels within the same season.

Baseline characteristics grouped by serum 25-OHD groups in women and men are shown in Table 1 and Table S1 and are all matched. In women, the median of normal fertilisation rates were 62.50, 64.29, and 68.75%, respectively (*P* = 0.001); with significant differences between Groups A and B, Groups A and C, Groups B and C (*P* = 0.043, 0.000, and 0.032, respectively). However, we observed no significant differences in men among the groups (*P* = 0.338).

**Table 1:** Baseline characteristics grouped by serum 25-OHD groups in women

Characteristics	Group A	Group B	Group C	*P* value
Number of cycles	232	768	232	
25-OHD in women (ng/ml)	10.26 ± 2.29	19.47 ± 3.52	31.13 ± 5.25	.000
Female age (y)	30.50 ± 4.09	30.61 ± 4.31	30.30 ± 4.38	.620
Male age (y)	32.63 ± 5.06	32.28 ± 5.22	32.23 ± 5.28	.628
Baseline FSH (mIU/ml)	6.84 ± 1.12	6.97 ± 1.18	6.89 ± 1.19	.278
BMI (kg/m^2^)	21.58 ± 2.71	21.65 ± 2.39	21.39 ± 2.61	.355
Previous pregnancy				.950
No	142 (61.2%)	432 (56.3%)	124 (53.2%)	.211
Yes	90 (38.8%)	136 (43.7%)	108 (46.8%)	
Duration of infertility (y)	3.62 ± 2.37	3.34 ± 2.59	3.25 ± 2.68	.253
COH protocol				.158
Long protocol	134 (57.8%)	431 (56.2%)	122 (52.4%)	
Prolonged protocol	37 (15.9%)	160 (20.9%)	59 (25.3%)	
Antagonist protocol	61 (26.3%)	177 (22.9%)	51 (22.3%)	
Duration of Gn treatment (days)	9.92 ± 1.39	10.11 ± 1.57	10.18 ± 1.70	.416
Dose of gonadotrophins (IU)	2167.69 ± 536.18	2172.84 ± 715.73	2125.27 ± 741.07	.665
Number of oocytes retrieved	13.00 ± 5.68	13.65 ± 6.39	14.09 ± 6.58	.167
Number of MII oocytes	11.72 ± 5.36	12.43 ± 5.88	12.98 ± 6.20	.064
Normal fertilization rate (%)	62.50 (50.00,71.43)^*^	64.29 (50.00,76.52)^*^	68.75 (53.33,80.00)^*^	.001

Data are expressed as mean ± SD or Number (Percentage) or median (interquartile range). Serum 25-OHD levels in women were classified into three groups: < 13.3 ng/ml (Group A),13.3–25.9 ng/ml (Group B), > 25.9 ng/ml (Group C). The symbol "*" meant that the median of normal fertilisation rates in women were 62.50%, 64.29%, and 68.75%, respectively (*P* = 0.001); with significant differences between Groups A and B, Groups A and C, Groups B and C (*P* = 0.043, 0.000, and 0.032, respectively). Abbreviations: 25-hydroxyvitaminD (25-OHD); Follicle-stimulating hormone (FSH); Body mass index (BMI); Controlled ovarian hyperstimulation (COH); Gonadotrophins (Gn); Metaphase II (MII)

### Serum 25-OHD levels in couples and normal fertilisation rates

Table 2 shows the generalized linear regression results of serum 25-OHD levels in couples and normal fertilisation rates, adjusted for female age, sperm progressive motility rate, and so on. Table 3 shows the marginal mean of the normal fertilisation rate in the female and male 25-OHD groups after adjusting for confounding factors in the model. The female 25-OHD group, pregnancy history (*P* = 0.042), number of oocytes obtained (*P* = 0.006), and daily dose of gonadotrophins administered (*P* = 0.021) were related to normal fertilisation rate. Compared with Group A in the female 25-OHD group, the ORs were 1.054 in Group B (*P* = 0.044; 95% CI, 1.001–1.110), and 1.111 in Group C (*P* = 0.002, 95% CI, 1.041–1.187). After adjusting for female age, BMI, baseline FSH, duration of infertility, daily dose of gonadotrophins administered, and number of mature oocytes retrieved, the marginal mean of normal fertilisation rate for Groups A, to B, and C were 59.50, 62.72 and 66.13%, respectively (*P* = 0.007). To analyse the relationship between female 25-OHD and normal fertilisation rate, female and male 25-OHD levels were taken as continuous variables; and we observed a linear association with normal fertilisation rates via Spearman’s correlation test (r = 0.101, *P* < 0.001). Male 25-OHD groups were not significantly associated with normal fertilisation rate(r = 0.045, *P* = 0.117). The marginal means of normal fertilisation rate in Groups A, to B, and C in men were 62.70, 63.00, and 62.30%, respectively (*P* = 0.903).

**Table 2:** Generalized linear regression analysis among all covariates and normal fertilisation rate

	OR	95%CI lower limit	95%CI upper limit	*P* value
Male 25-OHD ^a^				
Group A	REF			
Group B	1.005	0.955	1.058	.854
Group C	0.994	0.931	1.061	.847
Primary infertility				
Yes	0.960	0.923	0.998	.042
No	REF			
Stimulation protocol				
GnRH-ant	0.958	0.915	1.044	.072
GnRH-a prolonged	0.974	0.927	1.023	.290
GnRH-a long	REF			
Female 25-OHD ^b^				
Group A	REF			
Group B	1.054	1.001	1.110	.044
Group C	1.111	1.041	1.187	.002
BMI (kg/m2)	0.996	0.989	1.003	.301
Female age (y)	1.002	0.995	1.008	.624
Male age (y)	1.000	0.994	1.005	.971
PR (%)	1.001	0.999	1.002	.246
Baseline FSH (mIU/ml)	1.010	0.993	1.026	.255
Duration of infertility (y)	0.994	0.986	1.001	.114
Number of oocytes retrieved	0.996	0.992	0.999	.006
Daily dose of Gn (IU)	0.974	0.953	0.996	.021

Table 2 depicts the generalized linear regression results of serum 25-OHD levels in couples and normal fertilisation rates. ^a^ Serum 25-OHD levels in men were classified into three groups: < 16.3 ng/ml (Group A), 16.3–32.8 ng/ml (Group B), > 32.8 ng/ml (Group C). ^b^ Serum 25-OHD levels in women were classified into three groups: < 13.3 ng/ml (Group A),13.3–25.9 ng/ml (Group B), > 25.9 ng/ml (Group C). Abbreviations: Sperm progressive motile sperm rate (PR)

**Table 3:** Marginal mean of normal fertilisation rate by generalized linear regression

Group	Normal fertilisation rate(%)	
	Unadjusted^a^	Adjusted^b^
Female 25-OHD (ng/ml)		
0–13.2 (10.26)	0.593 ± 0.201	0.595 (0.567,0.623)
13.3–25.9 (19.40)	0.626 ± 0.205	0.627 (0.609,0.645)
> 25.9 (29.80)	0.658 ± 0.191	0.661 (0.631,0.691)
P^c^	0.002	0.007
Male 25-OHD (ng/ml)		
0–16.2 (13.10)	0.614 ± 0.191	0.626 (0.597,0.655)
16.3–32.8 (23.70)	0.627 ± 0.204	0.631 (0.613,0.649)
> 32.8 (37.30)	0.634 ± 0.205	0.625 (0.596,0.653)
P^c^	0.542	0.903

^a^ Values are mean ± SD

^b^ Values are marginal means (95% CIs) adjusted for female age, baseline FSH, BMI, duration of infertility, daily dose of gonadotrophins, number of oocytes retrieved

^c^ Values were obtained from x^2^ test (based on the marginal mean of estimation)

### Low fertilisation rates and 25-OHD groups in men and women

Table 4 shows our binary multivariate logistic regression between low fertilisation rates and 25-OHD groups. Table 5 shows the marginal mean of low fertilisation rate in the female and male 25-OHD groups after adjusting for confounding factors based on our model. Low fertilisation rate was defined as less than 20% of normal fertilisation rate. We found that only female 25-OHD groups were correlated with low fertilisation rate: compared with Group C, the ORs for exhibiting low fertilisation rate were 4.814 in Group A (95% CI, 1.266–18.309, *P* = 0.021), 3.204 in Group B (95% CI, 0.949–10.812, *P* = 0.061). Marginal means of the three groups were 0.05, 0.03, and 0.01, respectively (*P* = 0.022). In contrast, we observed no significant differences in the probability of low fertilisation rate between different male 25-OHD groups (*P* > 0.05); marginal means of the three groups were 0.02, 0.03, and 0.03, respectively (*P* > 0.05).

**Table 4:** Binary logistic regression analysis between 25-OHD quartiles and low fertilisation rate

	OR	95%CI	*P* value
Female 25-OHD group			
Group A	4.814	1.266, 18.309	0.020^*^
Group B	3.204	0.949, 10.812	0.061
Group C	REF		
Male 25-OHD group			
Group A	0.520	0.164, 1.643	0.265
Group B	0.981	0.431, 2.235	0.964
Group C	REF		
Female age (y)	0.953	0.860, 1.058	0.953
Male age (y)	1.050	0.968, 1.140	0.238
PR (%)	1.001	0.979, 1.024	0.924
Baseline FSH (mIU/ml)	1.006	0.766, 1.322	0.965
Primary infertility			
No	REF		
Yes	1.023	0.542, 1.932	0.944
Duration of infertility (y)	1.084	0.977, 1.202	0.130
BMI (kg/m2)	1.044	0.933, 1.168	0.455
Stimulation protocol			
GnRH-ant	REF		
GnRH-a prolonged	1.324	0.563, 3.116	0.520
GnRH-a long	1.502	0.570, 3.955	0.410
Daily dose of gonadotrophins (IU)	0.920	0.644, 1.315	0.648
Number of oocytes retrieved	0.946	0.891, 1.004	0.067

Table 4 depicts the binary logistic regression analysis results of serum 25-OHD levels in couples and normal fertilisation rates (LFR). Normal fertilisation rate > 0.2 (without LFR) was set as response, and normal fertilisation rate < 0.2 (with LFR) was set as reference. The symbol "*" meant that the ORs for exhibiting low fertilisation rate were 4.814 in Group A (95% CI, 1.266–18.309, *P* = 0.021) compared with Group C

**Table 5:** Marginal mean of low fertilisation rate by binary logistic regression

Group	Low fertilisation rate	
	Unadjusted^a^	Adjusted^b^
Female 25-OHD (ng/ml)		
0–13.2 (10.26)	0.05 ± 0.22	0.05 (0.02, 0.07)
13.3–25.9 (19.40)	0.04 ± 0.19	0.03 (0.02, 0.05)
> 25.9 (29.80)	0.01 ± 0.11	0.01 (0.00, 0.02)
P	0.07	0.02
Male 25-OHD (ng/ml)		
0–16.2 (13.10)	0.03 ± 0.16	0.02 (0.00, 0.03)
16.3–32.8 (23.70)	0.04 ± 0.20	0.03 (0.01, 0.04)
> 32.8 (37.30)	0.03 ± 0.18	0.03 (0.01, 0.05)
P	0.57	0.29

^a^ Values are mean ± SD

^b^ Values are marginal means (95% CIs) adjusted for female age, baseline FSH, BMI, duration of infertility, daily dose of Gn, number of oocytes retrieved

## Discussion

Normal fertilisation is one of the most important steps limiting for IVF success, but IVF provides a unique opportunity to explore this problem because it is possible precisely to track follicular development, oocyte fertilisation, embryonic development, and clinical outcome. Since the quality of the oocyte and fertilising ability of sperm are both essential for normal fertilisation, we measured serum vitamin D levels of couples prior to COH. In this prospective observational and large-sample study (1232 couples), the marginal means of the normal fertilisation rate from Group A, to B, and C in women were 59.50, 62.72, and 66.13%, respectively (*P* = 0.007). After binary logistic regression analysis of female groups, compared with Group C, the ORs for exhibiting a low fertilisation rate were 4.814 in Group A (95% CI, 1.266–18.309, *P* = 0.021), 3.204 in Group B (95% CI, 0.949–10.812, *P* = 0.061).

These results are supported by the fundamental research reported by Abadia et al., who demonstrated that the adjusted fertilisation rates for women in increasing quartiles of serum 25-OHD were 0.62 (95% CI, 0.51–0.72), 0.53 (95% CI, 0.43–0.63), 0.67 (95% CI, 0.56–0.76), and 0.73 (95% CI, 0.63–0.80), respectively (*P*-trend = 0.03) [25]. But the Abadia study only included 100 women (25 per group), and most of the participants were White/Caucasian. Another study that supported our findings was was one where 221 infertile women underwent an IVF cycle, and deficient, insufficient, and sufficient levels of vitamin D were defined as 10, 10–29, and 30–100 ng/ml, respectively. These authors observed a trend towards increased fertilisation (43.17, 53.37, and 58.77%; *P* = 0.054) [26]. In contradistinction, investigators in two other reports noted that the serum levels of 25-OHD in women correlated negatively with the oocyte’s ability to undergo fertilisation in ICSI cycles [27, 28]. Their possible explanation was that as the fertilisation process involves a multi-step, multi-component interaction between oocyte and sperm, vitamin D deficiency in women may have affected the process of sperm entering the oocyte.

Successful fertilisation involves not only the quality of the oocyte but also the fertilizing ability of the sperm. VDR was shown to be expressed in neck and post-acrosomal regions of spermatozoa, and found to be higher in normal men than in infertile men [29]. Sperm-function tests indicated that follicular concentrations of 1,25(OH)_2_D_3_ induced a modest increase in calcium influx in spermatozoa and sperm-oocyte binding in vitro [14, 29]. Two groups have found that 1,25(OH)_2_D_3_ increased intracellular calcium in human sperm and promoted sperm motility and the acrosome reaction *in vitro* [20, 21], suggesting that vitamin D plays an important role in the fertilizing ability of sperm. Ours, however, is the first report on the correlation between male vitamin D and fertilisation rate in IVF cycles. Nonetheless we failed to find an association between normal fertilisation rate or LFR and serum 25-OHD in men undergoing IVF, using either Pearson’s correlation or in binary logistic regression analysis. The possible explanation is that the threshold for normal sperm fertilisation in men is relatively low, and research at high alatitudes is therefore warranted. Another possible reason is that 25-OHD in the fertilisation medium may have partially compensated for the deficiency in 25-OHD in male serum.

The present study exhibits several strengths. First, our study possessed the largest sample size studied thus far in this research area. Previous studies only included 220, 100, 198, and 82 female patients respectively [25–28]. Second, we are the first group to evaluate the relationship between serum 25-OHD of couples and fertilisation rate. Third, to avoid seasonal differences, we only included data from those couples where the time-intervalbetween serum 25-OHD measurement and ovum pick-up was still within the same season. And lastly, according to the Institute of Medicine, a sufficient vitamin D level is defined as higher than 20 ng/ml, so as to avoid adverse skeletal damage. However, as the threshold may not fit for reproductive health in men and women, we classified the levels of 25-OHD into three groups: Group A, less than 10%; Group B, 10 to 90%; and Group C, above 90%. We then ultimately explored the statistical correlation between 25-OHD levels and fertilisation rate by using generalized linear regression and binary logistic regression.

We also need to address several limitations to our study. First, this was an observational study and consequently demonstrated an association-but not causation with regard to the relationship between vitamin D levels and biologic outcomes. Demonstration of causation will require new studies that entail vitamin D supplementation. Second, we we did not measure vitamin D binding protein (VDBP). Therefore, we consider only total 25-OHD, even though bioavailable 25-OHD is defined as circulating 25-OHD not bound to VDBP. Third, different human races display various VDBP genetic polymorphisms, which affect the affinity between VDBP and 25-OHD. Blacks and Asians manifest the highest affinity for 25-OHD, which is associated with low VDBP levels. Thus levels of total 25-OHD are, in part, genetically determined [30]; but our study is based on East Asians. Besides, 25-OHD in follicular fluid, which had a strong positive correlation with the concentration of vitamin D in serum [31], might have a greater role in maintaining oocyte quality and associate with top quality embryo compared with 25-OHD in serum [32]. In contrast, our study was not designed to evaluate local-intrafollicular vitamin D level. It is worth to identify further whether local vitamin D (in the follicular fluid) or systemic vitamin D matters. Finally, 1,25-hydroxycholecalciferol, the active form of vitamin D, binds and activates vitamin D receptor. Reginatto MW, et al. showed that the vitamin D receptor taql polymorphism associated with a reduced number of follicles in women [33]. Therefore it is important that the relationship of vitamin D receptors polymorphism and normal fertilization needs to be studied in the future.

In conclusion, our study demonstrated that serum 25-OHD concentrations in women(but not in men) in couples undergoing IVF were positively correlated with normal fertilisation rates. We observed that a lower serum vitamin D level in women was a high risk factor for a low fertilisation rate in IVF cycles. Clearly, the establishment of an optimal threshold for vitamin D levels in human reproduction requires further elucidation. Studies of vitamin D supplementation are also needed to clarify causality between low fertilisation rates and vitamin D deficiency in women.

## Conclusions

In summary, we found that circulating 25-OHD concentrations in women appear to be associated with normal fertilisation rates and low fertilisation rates in IVF cycles, but not in men. A further randomized controlled trial with vitamin D supplementation is needed to demonstrate whether female vitamin D levels exert an effect on the normal fertilisation process before vitamin D supplementation can be recommended to bring potential fertility benefits.

## Supplementary information

**Additional file 1: Table S1.** Baseline characteristics grouped by serum 25-OHD groups in men. The mean 25-OHD levels in each month among men and women were illustrated in Fig. S1 and S2, by which we classified 12 months into four seasons: June to Octobor; November; December to April; and May. There were no statistical differences among mean 25-OHD levels in the same season.

## Data Availability

The datasets used and/or analysed during the current study are available from the corresponding author on reasonable request.

## Abbreviations

IVF: In-vitro fertilisation
25-OHD: 25-hydroxyvitaminD
LFR: Low fertilisation rate
VDR: Vitamin D receptor
FSH: Follicle-stimulating hormone
BMI: Body mass index
GnRH-a: Gonadotropin releasing hormone agonist
E2: Estradiol
LH: Luteinizing hormone
GnRH-ant: Gonadotropin releasing hormone antagonist
HCG: Human chorionic gonadotropin
COH: Controlled ovarian hyperstimulation
VDBP: Vitamin D binding protein
PR: Sperm progressive motile sperm rate
Gn: Gonadotrophins
MII: Metaphase II
