# Supplementary material for: Normal fertilisation rates and serum 25-OHD levels among couples undergoing in-vitro fertilisation: a prospective cohort study
Source: BMC Pregnancy Childbirth. 2020 Jun 5;20:346. doi: 10.1186/s12884-020-02959-z (PMC7275449; doi:10.1186/s12884-020-02959-z)
Supplement: Supplementary file 1 — Additional file 1: Table S1. Baseline characteristics grouped by serum 25-OHD groups in men. The mean 25-OHD levels in each month among men and women were illustrated in Fig. S1 and S2, by which we classified 12 months into four seasons: June to Octobor; November; December to April; and May. There were no statistical differences among mean 25-OHD levels in the same season. [file 12884_2020_2959_MOESM1_ESM.docx]

**Supplemental Table 1 Baseline characteristics grouped by serum 25-OHD groups in men.**

| Characteristics Group A Group B Group C *P* value | | | | | |  |  |
| --- | --- | --- | --- | --- | --- | --- | --- |
| Number of cycles | 232 | 768 | 232 |  |  |  |  |
| 25-OHD in men (ng/ml) | 12.69±2.55 | 23.98±4.68 | 38.90±5.27 | .000 |  |  |  |
| Female age (y) | 30.84±3.80 | 30.48±4.24 | 30.40±4.83 | .455 | | |  |
| Male age (y) | 32.48±4.91 | 32.26±5.06 | 32.44±5.92 | .799 | | |  |
| Baseline FSH (mIU/ml) | 6.81±1.22 | 6.98±1.16 | 6.91±1.13 | .158 | | |  |
| BMI (kg/m^2^) | 21.47±2.55 | 21.63±2.61 | 21.63±2.65 | .717 | | |  |
| Previous pregnancy |  |  |  | .387 | | |  |
| No | 138(59.5%) | 423(55.1%） | 137(58.8%) |  | | |  |
| Yes | 94(40.5%） | 345(44.9%) | 95(41.2%) |  | | |  |
| Duration of infertility (y) | 3.38±2.54 | 3.36±2.51 | 3.43±2.79 | .934 | | |  |
| COH protocol  Long protocol  Prolonged protocol  Antagonist protocol | 137(59.1%)  33(14.2%)  62(26.7%) | 427(55.7%)  164(21.4%)  177(22.9%) | 123(2.8%)  59(25.3%)  50(21.9%) | .053 | | |  |
| Duration of Gn treatment (days) | 9.96±1.45 | 10.14±1.76 | 9.98±1.48 | .196 | | |  |
| Dose of gonadotropins (IU) | 2114.52±697.33 | 2169.64±696.18 | 2188.7±763.83 | .483 | | |  |
| Number of oocytes retrieved | 14.00±6.53 | 13.56±6.12 | 13.39±6.69 | .545 | | |  |
| Number of MII oocytes | 12.68±6.06 | 12.37±5.68 | 12.25±6.24 | .703 | | |  |
| Normal fertilization rate (%) | 62.50(50.00,75.00) | 64.29(50.00.76.92) | 66.67(50.00,77.78) | .338 | | |  |

.Data are expressed as mean ± SD or Number (Percentage) or median (interquartile range).

The mean 25-OHD levels in each month among men and women were illustrated

in Figure S1 and S2, by which we classified twelve months into four seasons: June to Octobor; November; December to April; and May. There were no statistical differences among mean 25-OHD levels in the same season.
